# DICER1 in the Pathogenesis of Age-related Macular Degeneration (AMD) - *Alu* RNA Accumulation versus miRNA Dysregulation

**DOI:** 10.14336/AD.2019.0809

**Published:** 2020-07-23

**Authors:** Kai Kaarniranta, Elzbieta Pawlowska, Joanna Szczepanska, Janusz Blasiak

**Affiliations:** ^1^Department of Ophthalmology, University of Eastern Finland, Kuopio 70211, Finland and Department of Ophthalmology, Kuopio University Hospital, Kuopio 70029, Finland.; ^2^Department of Orthodontics, Medical University of Lodz, 92-216 Lodz, Poland.; ^3^Department of Pediatric Dentistry, Medical University of Lodz, 92-216 Lodz, Poland.; ^4^Department of Molecular Genetics, Faculty of Biology and Environmental Protection, University of Lodz, 90-236 Lodz, Poland.

**Keywords:** age-related macular degeneration, DICER1, *Alu* repeats, miRNA regulation, NLRP3 inflammasome

## Abstract

DICER1 deficiency in the retinal pigment epithelium (RPE) was associated with the accumulation of *Alu* transcripts and implicated in geographic atrophy (GA), a form of age-related macular degeneration (AMD), an eye disease leading to blindness in millions of people. Although the exact mechanism of this association is not fully known, the activation of the NLRP3 inflammasome, maturation of caspase-1 and disruption in mitochondrial homeostasis in RPE cells were shown as critical for it. DICER1 deficiency results in dysregulation of miRNAs and changes in the expression of many genes important for RPE homeostasis, which may also contribute to AMD. DICER1 deficiency can change the functions of the miR-183/96/182 cluster that regulates photoreceptors and their synaptic transmission. Aging, the main AMD risk factor, is associated with decreased expression of DICER1 and changes in its diurnal pattern that are not synchronized with circadian regulation in the retina. The initial insult inducing DICER1 deficiency in AMD may be oxidative stress, another major risk factor of AMD, but further studies on the role of deficient DICER1 in AMD pathogenesis and its therapeutic potential are needed.

Age-related macular degeneration (AMD) is emerging as a major cause of global vision loss in the elderly in the developed countries. AMD is characterized by the degeneration of retinal pigment epithelium (RPE) located between photoreceptors and the choriocapillaris. Degeneration of RPE cells evokes an irreversible dysfunction of photoreceptors and eventually vision loss [1]. AMD is frequently divided into two basic forms: dry (non-exudative) and wet (exudative, neovascular) with the former representing an atrophic, whereas the latter reveals a neovascular disease phenotype. In its early stage, AMD is characterized by changes in RPE pigmentation, accumulation of lysosomal lipofuscin and the presence of extracellular yellowish drusen deposits. Accumulation of lipofuscin and drusen is a hallmark of AMD progression [2].

In its advanced form, dry AMD is characterized by the presence of clearly distinguishable atrophic lesions of the outer retina resulting from the loss of RPE cells and photoreceptors (Fig. 1). These lesions, which look like islands surrounded by a sea of normal retina in autofluorescence imaging, define the presence of geographic atrophy (GA), an advanced form of dry AMD [3]. At present, there is no remedy for GA and therefore molecular studies to understand its pathogenesis are justified.

**Figure 1.**
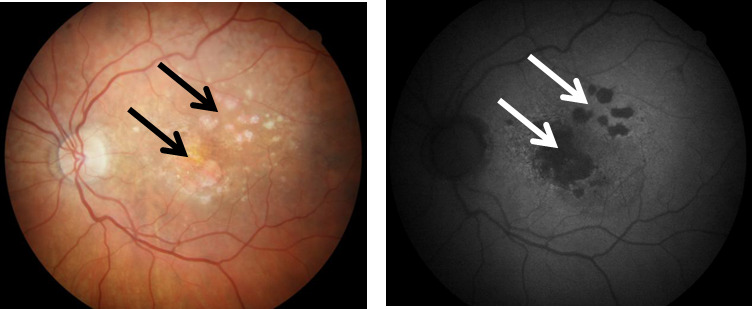
**Color fundus photograph (left) and fundus autofluorescence (FAF, right) of a patient with geographic atrophy (GA), an advanced form of age-related macular degeneration**. Arrows indicate GA lesions with irreversibly damaged photoreceptors and retinal pigment epithelial cells. Increased FAF is detected around GA lesions.

The order of pathogenic events leading to macular degeneration may well be different in the two forms of AMD [1]. In dry AMD, the initial insult may originate from impaired proteostasis that leads to lipofuscin and drusen formation and increased inflammation. Dry AMD can shift to wet AMD that is closely associated with the loss of choroidal vessels and hypoxia and subsequent choroidal neovascularization. It is not known why one individual develops dry AMD, while another suffers from its wet counterpart.

AMD is a complex disease with genetic, environmental and life-style factors contributing to its pathogenesis [1]. Mutations in the *complement factor H* and *ARMS2/HTRA1* genes are considered as the major genetic factors in AMD pathogenesis, but variations in genes involved in cellular antioxidant defense, including DNA repair genes and their regulators are also frequently associated with AMD [4, 5]. Several other genes, including those involved in autophagy, fat and xenobiotics metabolism have been reported to be important in AMD [6-8]. The majority of studies on the role of genetic factors in AMD pathogenesis have focused on the variability in the sequence of candidate genes. However, it is not only the sequence of a gene, but also its epigenetic profile which determines the role played by its product, including conferring a pathological phenotype. However, epigenetic mechanisms in AMD are poorly understood [9-12]. The emerging role of micro RNA (miRNA) in gene regulation is also reflected in AMD, but the exact involvement of miRNAs in AMD pathogenesis is far from clear [13].

Chronic inflammation is frequently reported to associate with AMD and the activation of the NLRP3 (NACHT (neuronal apoptosis inhibitor protein, class 2 transcription activator of the MHC, heterokaryon incompatibility and telomerase-associated protein 1), NLR (nucleotide-binding domain, leucine-rich repeat-containing family), and PYD (pyrin domain)-containing protein 3) inflammasome is considered as an important mechanism in AMD pathogenesis (reviewed in [14]). Activated NLRP3 is responsible for the maturation of caspase-1 via the induction of its autocleavage, resulting in the release of proinflammatory cytokines such as interleukin-1 beta (IL-1β) and IL-18 [15]. NLRP3 is a member of the inflammasome family containing an apoptosis-associated speck-like protein with a caspase recruitment domain (ASC). NLRP3 activation is preceded by its priming, usually requiring an involvement of the NF-κB (nuclear factor kappa B) pathway and prointerleukin-1β (pro-IL-1β) transcription [16]. However, pro-IL-1β is not constitutively expressed in RPE cells and the endogenous levels of NLRP3 components are too low to activate the inflammasome and therefore its priming is critical in RPE cells [17, 18].

The activation of NLRP3 and the release of caspase-1 have been claimed to be involved in cell death in atrophic AMD models. Moreover, the activation of the inflammasome was revealed as a critical step in the degeneration of RPE cells induced by the accumulation of double-stranded (ds) RNA transcripts of the *Alu* transpozons, the most common mobile elements in the human genome [19]. The accumulation of *Alu* RNA resulted from the impairment of the DICER1 endoribonuclease, which is involved in cleavage of various dsRNAs, including precursors of miRNAs. This review presents evidence for the involvement of DICER1 impairment, resulting in *Alu* accumulation in RPE cells and thus in the pathogenesis of AMD. We also describe an alternative pathway linked with DICER1 impairment - dysregulation of miRNAs which would normally be important for homeostasis in RPE cells.

## DICER1 is an endoribonuclease involved in miRNA biogenesis and the regulation of *Alu* transposons

DICER is a protein belonging to the ribonuclease (RNase) III family that plays an important role in the biogenesis of small RNAs, including micro RNA (miRNA) and short interfering RNA (siRNA). Humans have one DICER (DICER1), which functions in both miRNA and siRNA pathways [20]. In addition to DICER1’s basic function in miRNA biogenesis, it possesses other activities in processing small RNAs, which are typically 20-30 nt long, have 3’ single-stranded overhangs and canonical 3’ and 5’ ends. Furthermore, DICER1 has been reported to have additional functions not related to small RNA processing in both the cytoplasm and nucleus as well as in exosomes outside the cell [21]. DICER1 may be also involved in several other activities, e.g. autophagosome formation, passive RNA binding, control of nuclear pore components, antiviral control (reviewed in [22]).

*Alu* elements belong to the most common transposable elements (TEs) in the human genome with a copy number exceeding 1 million, which means that they make an over 10% contribution to the genome content. These elements do not encode any protein, but they acquire the factors needed for their propagation from members of the family of autonomous long interspersed nuclear elements 1 (LINE-1, L1) [23].

A typical *Alu* element is about 300 bp (base pair) and consists of two parts (arms) separated by an A-rich short stretch and tailored by a 3’ A-rich fragment. It has the A and B boxes of the internal RNA polymerase III promoter in which the transcription of the *Alu* element is initiated. Two enzymes are utilized to move *Alu* elements: an endonuclease and reverse transcriptase. The endonuclease cleaves a target site in the 5’-TTTT/AA-3’ sequence and the released consensus is bound by *Alu* RNA with its A-rich region. The synthesis of the cDNA first strand is initiated from a TTTT primer by L1 reverse transcriptase. A nick is made in the other strand of the target site and the second strand of the element is then synthesized by a host DNA polymerase.

In their propagation, *Alu* elements can be integrated into the coding part of the genome, resulting in mutations causing either gene inactivation or other detrimental changes. On the other hand, they can provide elements, such as enhancers, splice sites or even promoters, beneficial for the expression of genes nearby to the site of integration and thus increase human genetic diversity [24-26]. Moreover, changes in the genome induced by the expansion of *Alu* elements may also follow from their homologous recombination resulting in gene duplication, deletion and conversion [27]. These changes in the genome induced by the expansion of *Alu* elements have to be identified in many human diseases (reviewed in [28-31]).

In physiological conditions, *Alu* transcripts are kept at a low level, up to 1000 per cell, i.e. there must be a mechanism(s) controlling their propagation, and if these fail, there can be pathological consequences. This mechanism is far from clear, but it may include endonucleolytic processing of *Alu* transcripts by DICER1, as it has been reported that the DICER1-dependent degradation of *Alu* RNAs was critical for ameliorating detrimental effects in the human and mouse retinal cells [32].

## DICER1 impairment leads to *Alu* RNA accumulation inducing RPE degeneration in GA

**Figure 2.**
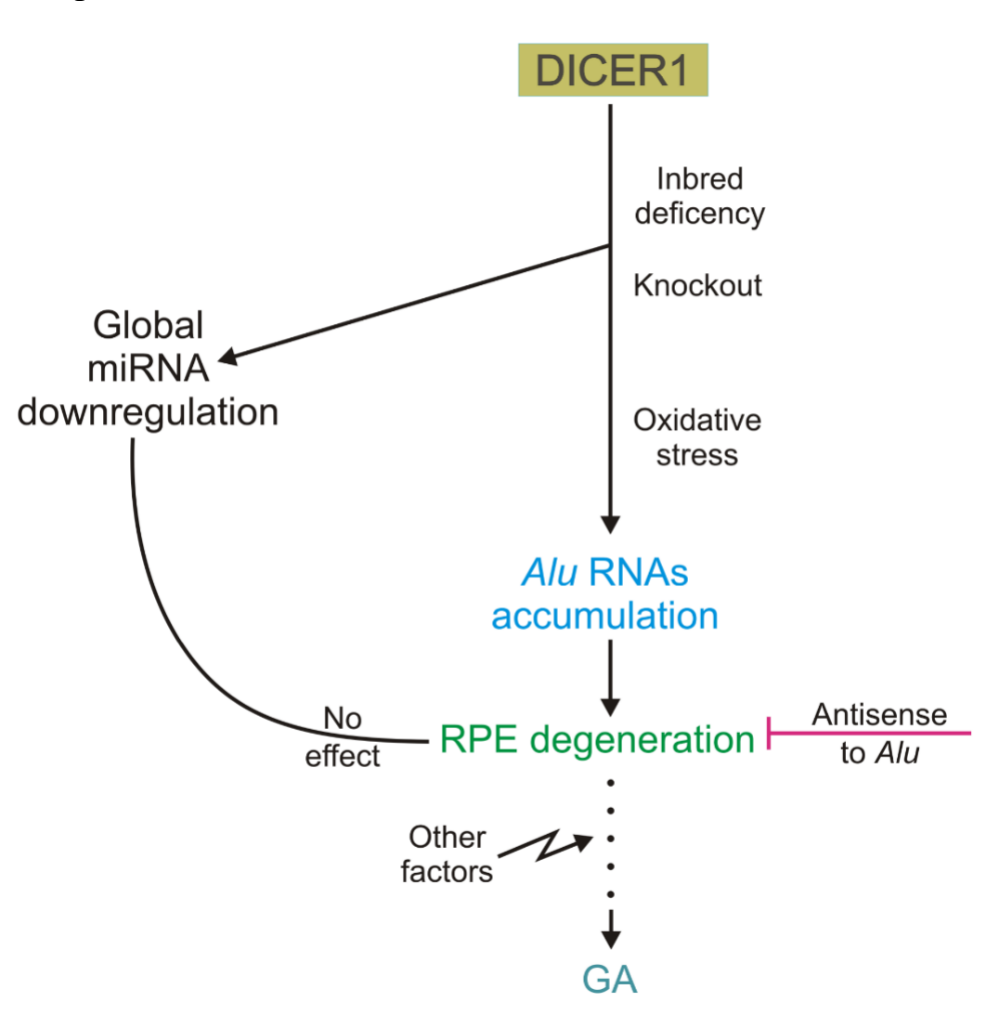
**Involvement of the DICER1 ribonuclease in the pathogenesis of geographic atrophy (GA), an incurable form of age-related macular degeneration (AMD)**. RNAs accumulate in animals with an inbred DICER1 deficiency as well as in human retinal pigment epithelium (RPE) cells with DICER1 knockout, and there is a global dysregulation of miRNAs. The same outcome can be observed when DICER1 is affected by oxidative stress. Independently of miRNA dysregulation, *Alu* RNAs accumulation can lead to RPE degeneration if this is inhibited by antisense oligonucleotides to *Alu* RNAs. Degeneration of RPE may be associated with the form of geographic atrophy (GA), an incurable type of age-related macular degeneration.

Kaneko et al. observed that there was reduced expression of DICER1 in RPE, but not in the neural retina in GA [32]. On the other hand, there was no decline in DICER1 in human RPE cells in other retinal diseases, such as retinitis pigmentosa, vitelliform macular dystrophy and retinal detachment nor was it evident in mouse models of retinal degeneration, suggesting that the DICER1 reduction evident in RPE in GA is not simply a common reaction to retinal injury [32]. There are DICER1-deficient strains of mice; these animals display an acquired RPE degeneration. Moreover, the increased death of human RPE cells was observed after antisense knockdown of the *DICER1* gene, which implies that DICER1 impairment may be involved in the pathogenesis of GA (Fig. 2).

GA, unlike many other retinal diseases, is associated with a decreased expression of DICER1 in retinal pigment epithelium (RPE) cells. It has been found that DICER1-deficient mouse strains acquired GA and human RPE cells with a knockout in the DICER 1 gene both displayed an elevated death ratio. The degeneration of RPE cells resulting from DICER1 depletion could be prevented by antisense oligonucleotides targeting *Alu* RNAs, but no such degeneration was observed after global miRNA downregulation. Therefore, DICER1 impairment may induce the accumulation of *Alu* RNAs that may contribute to the pathogenesis of GA.

In the search for the mechanism underlying the role of *DICER1* in GA, Kaneko et al. showed that the degeneration of RPE cells resulting from DICER1 depletion could be prevented by antisense oligonucleotides targeting *Alu* and *Alu-*like RNAs [32]. Similar effects were not observed in global miRNA downregulation. Therefore, this was interpreted as a novel, miRNA-independent cell survival function of DICER1. It seems that *Alu* RNAs accumulation can directly cause human pathology; this is a consequence of DICER1 deficiency which is not associated with an impairment in miRNA regulation, but instead should be linked with a DICER1-specific process [32]. The critical step in the search for the mechanisms underlying observed changes was the detection of an excess of dsRNAs which were about 300 nt long in RPE of human DICER1-deficient eyes affected by GA. These RNAs exhibited a high homology to the consensus sequence of the *Alu* Sq subfamily and were not present in the reference genome. In their work, Kaneko et al. observed that *Alu* RNA from RPE of GA-affected eyes was approximately 300 nucleotides long suggesting that it could be a transcript of RNA polymerase III [32]. However, extensive RPE degeneration was not observed in adult mice deficient in genes critical for miRNA pathways, such as Drosha, Dgcr1, Tarbp2 and Ago1/2/3/4 [32].

In searching for the initial factor triggering RPE-specific reduction of DICER1, Kaneko et al. observed that hydrogen peroxide downregulated the DICER1 gene in human RPE cells [32]. As H_2_O_2_ is a model factor to induce oxidative stress in cell cultures, they suggested that this stress, which is a recognized factor in AMD pathogenesis, can initiate a cascade of events leading to accumulation of *Alu* transcripts.

Taken together, oxidative stress can induce DICER1 impairment in RPE, leading to an accumulation of *Alu* transcripts and eventually to RPE degeneration and GA. However, there are two important questions to be considered: (1) how does an excess of *Alu* RNA induce RPE degeneration and (2) why is this effect specific for GA and not for other degenerative retinal disorders?

**Figure 3.**
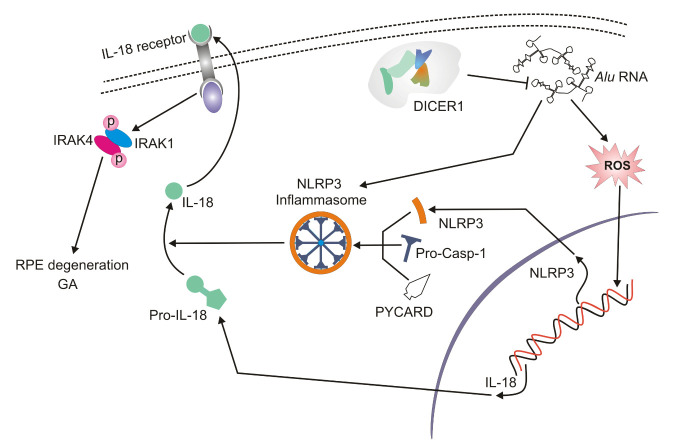
**The accumulation of *Alu* RNAs leads to inflammasome formation and activation of interleukin 18 (IL-18) both of which may contribute to geographic atrophy (GA)**. DICER1 deficiency results in an excess of *Alu* RNAs; these induce oxidative stress and increase the production of reactive oxygen species (ROS). Subsequently, ROS prime the mRNA of *NLRP3* (NACHT, LRR and PYD domains-containing protein 3) and *IL18.* NLRP3 associates with PYCARD and procaspase 1 to form the NLRP3 inflammasome that converts pre-interleukin 8 into its mature form, which in turn, mediates the activation of IRAK1 and IRAK4 (interleukin-1 receptor-associated kinase 1 and 4) that contribute to RPE cells death, RPE degeneration and eventually GA.

## NLRP3 inflammasome is the critical connection between accumulating *Alu* RNAs and the degenerating RPE

Tarallo et al. reported that *Alu* RNA overexpression mediated by DICER1 deficiency in RPE cells activated caspase-1 and the MyD88 (myeloid differentiation primary response 88) protein (Fig. 3) [19]. The caspase activation took place within the NLRP3 inflammasome and MyD88 stimulation occurred independently of TLR (toll-like receptor) *via* IL-18 and its receptor. The *Alu* RNA excess primed the inflammasome through an overproduction of mitochondrial ROS. *Alu* RNAs can be toxic through several pathways, including the stimulation of the production of mitochondrial ROS via voltage-dependent anion channels (VDAC)-1/2. IL-18-mediated activation of MyD88 induced recruitment and phosphorylation of IRAK1 (interleukin-1 receptor-associated kinase 1) and IRAK4 and an excess of *Alu* RNA increased that phosphorylation. Thus, IRAK1/4 could induce RPE degeneration and thus participate in the route to GA (Fig. 3).

Further research revealed that an excess of *Alu* RNAs primed the NLRP3 inflammasome through a TLR-independent activation of NF-κB [33]. In addition, the P2X purinoreceptor 7 (P2X7) was a critical signaling intermediate in the NLRP3 priming evoked by *Alu* RNA excess. It was also shown that nucleoside reverse transcriptase inhibitors (NRTIs) could inhibit P2X7-mediated NLRP3 activation independently of reverse transcriptase inhibition, preventing the caspase-1 activation induced by *Alu* RNA [34].

It was also demonstrated that RPE degeneration in mouse eyes and human cell lines was associated with an increased phosphorylation of ERK1/2 (extracellular-signal-regulated kinase 1/2) [35]. This effect was also induced by either overexpression of *Alu* RNA or downregulation of DICER1 in both mouse RPE and human cell lines. Intravitreous administration of an inhibitor of MEK1 (mitogen-activated protein kinase kinase 1), an ERK1/2 activating kinase, diminished the extent of *Alu* RNA-induced degeneration of mouse RPE. Importantly, an increased level of phosphorylation of ERK1/2 was also observed in human eyes with GA. However, ERK1/2 activation effects on the regulation of cellular survival are cell-type and stress stimuli dependent [36, 37].

There is evidence that non-canonical activation of the NLRP3 inflammasome is a critical step in RPE degeneration and the development GA pathology attributable to a DICER1 deficiency resulting in *Alu* RNA accumulation [38]. This process involves the activation of caspases 4 (11 in mice) and 1 and depends on cyclic GMP-AMP synthase (cGAS)-dependent interferon-β (IFN-β) production and the secretion of gasdermin D-dependent interleukin-18 (IL-18). DICER1 deficiency/*Alu* RNA accumulation resulted in the release of mtDNA into the cytosol of RPE cells, which led to the activation of cGAS.

Dunaief et al. were the first to show that AMD eyes accumulated more iron than their non-AMD counterparts [39, 40]. Although iron toxicity in the retina is frequently associated with the catalysis of the Fenton reaction, resulting in ROS excess, the complete mechanism underlying the role of iron in AMD pathogenesis is far from clear. Gelfand et al. showed that iron could induce the NLRP3 inflammasome signaling evoked by stimulation of *Alu* RNA accumulation [41]. These authors utilized both a mouse AMD model and human RPE cells and observed that iron overloading induced RPE degeneration, similar to that observed in human GA and resulted from NLRP3 activation in mouse models of AMD [19, 32, 34]. Caspase-1 maturation was also detected in that experiment, suggesting that iron excess could be considered as an inflammasome agonist in RPE cells. Inflammasome activation was not driven by other metals capable of catalyzing the Fenton reaction, although these metals were toxic to the RPE cell layer. Furthermore, antisense nucleotides to *Alu* RNA prevented the maturation of caspase-1. It was concluded that iron could lead to accumulation of *Alu* RNA resulting in inflammasome activation and retinal degeneration. Iron overload did not affect the level of transcription of the *DICER1* gene, but it did decrease DICER1 activity and this decrease resulted from the sequestration of poly(C)-binding protein 2 (PCBP2).

The mechanism of death of RPE cells in AMD is not completely clear although it is likely that more than one pathway of programmed cell death may be involved, depending on the form of the disease (reviewed in [42]). Kim et al. emphasized that the RPE cell death in GA linked with DICER1 deficiency/*Alu* accumulation was associated with the activation of caspase-8 through a mechanism, which depended on a Fas ligand [43].

## Impaired DICER1 can affect retina through miRNA dysregulation

Micro RNAs, which play an important role in the regulation of gene expression, are produced from the double-stranded region of an RNA hairpin precursor (pre-miRNA), which is formed by the cleavage of primary miRNA (pri-miRNA) transcribed from its gene, typically located in the introns of protein-coding genes or in an intergenic region. miRNA genes are transcribed by RNA polymerase II or III. Pri-miRNA in the nucleus folds into a hairpin structure and is processed by the microprocessor complex containing DGCR8 (DiGeorge syndrome critical region 8, Pasha) and the endoribonuclease III Drosha as well as other auxiliary proteins. This processing results in pre-miRNA, which is 60-70 nt long and has a 2 nt 3’ overhang. Pre-miRNAs are transported into the cytoplasm by the Ran-Exportin 5 complex with the involvement of GTP. Pre-miRNAs are cleaved in the cytoplasm by DICER1 complexed with TRBP (TAR RNA-binding protein), to produce short (19-25 nt) duplex miRNAs with 2 nt 3’ overhangs. TRBP recruits AGO2 (Argonaute 2) and other proteins of the Argonaute superfamily to assemble an RNA interference silencing complex (RISC), containing several other proteins. miRNAs are denatured and one strand (the guide strand) is assembled into the RISC complex, while the other strand (the passenger strand, usually denoted as miRNA*) is degraded [44]. Mature miRNAs can modify translation by decreasing the number of target mRNAs and inhibiting the movement of the ribosome.

Around 100 miRNAs have been identified in the mouse retina and over 20 of them are retina-specific. miRNAs have different and partly overlapping patterns of expression in different tissues e.g. in the cells of mammalian eye, in particular in corneal epithelium, lens and retina [45]. miRNAs are specifically expressed in the developing and adult retina, but their specificity in aging adult retina is a matter of debate. Georgi and Reh screened many miRNAs in the mouse developing retina by conditional knockout (CKO) of DICER1 and concluded that miRNAs can regulate both the competence of retinal progenitor cells and the differentiation and maturation of retinal neurons [46]. DICER1 and miRNAs can be important for the differentiation of stem cells.

Pinter et al. observed that the conditional deletion of DICER in the developing mouse retina resulted in a decrease in several miRNAs and perturbations in the visual system, including a significant reduction of the size of the eye [47]. These authors also observed an increased apoptosis in the retina and defects in the structure of the optic fiber.

In addition to choroidal neovascularization in wet AMD formation of new retinal vessel may be observed in ischemic proliferative diabetic retinopathy and vein occlusion [48]. Shen et al. observed down- and up-regulation of various miRNAs in mouse ischemic retinas displayed the features of wet AMD, including neovascularization [49]. Since anti-VEGF (vascular endothelial growth factor) strategy is virtually the only remedy in wet AMD, its efficacy can be assisted by modulation of the expression of miRNAs regulating VEGF or other proteins involved in angiogenesis. Shen et al. postulated that the decrease in VEGF expression might result from the reduction of HIF-1α (hypoxia inducible factor 1 alpha subunit) induced by miR-31 [49]. An association of miRNAs with neovascularization, which is the main pathogenic effect in wet AMD, can help in the identification of new genes/proteins that are involved in this process as new therapeutic targets. Many miRNAs were reported to be dysregulated in AMD and they are involved in processes and effects of AMD pathogenesis: inflammation, oxidative stress, angiogenesis and others (reviewed in [50]).

miRNAs play an important role in photoreceptors that are constantly challenged by various stress factors, mostly light exposure and the high level of oxygen and therefore they must precisely regulate the expression of their genes to cope with such detrimental conditions [51-56]. Busskamp et al. demonstrated that miR-182 and miR-183 are essential for proper visual functions as their specific loss in cones underlined by disruption of DGCR8 resulted in a decreased response to light [51]. DICER1 was identified as an element of this regulation [54]. As RPE cells are adjacent to photoreceptors and phagocytose the outermost parts of photoreceptor outer segments, they are also directly or indirectly challenged by stress factors affecting photoreceptors. In general, results from Ambati’s group suggest that the *Alu* RNA accumulation due to DICER1 deficiency plays a role in AMD pathogenesis independently of disturbances in miRNA processing associated with impairment DICER1. Sundermeier et al. suggested that a deficiency in miRNA biogenesis resulting from the loss of DICER1 activity could also contribute to the death of RPE cells observed in GA [55]. These authors disrupted two essential and independent steps in miRNA biogenesis depending on *DGCR8*, a cofactor for the DROSHA endoribonuclease, specific for dsRNA and *DICER1* in RPE cells of mice exhibiting the doxycycline-inducible expression of the *Cre* (CAMP (cyclic adenosine 3,5-monophosphate) response element) recombinase. These results suggest that the DICER1 deficiency can be associated with the pathogenesis of GA not only by a toxic accumulation of *Alu* RNAs, but also by a loss of miRNAs regulating the expression of essential genes in RPE cells. In their earlier work, Sundermeier et al. observed that the conditional deletion of DICER1 in mature mouse rods led to retinal degeneration and the loss of visual functions [54]. They identified several miRNAs, including miR-22, miR-26, miR-92 and miR-124, which could be dysregulated by DICER1 deletion and contributed to the observed effects.

Retinal non-coding RNA 3 (Rncr3) is the main source of miR-124a, which plays a role in the development of the vertebrate central nervous system [56]. Sanuki et al. showed that miR-124a prevented apoptosis in the developing mouse retina, being essential for the maturation of retinal cones [57]. This effect was associated with a translational silencing of the *Lnx2* (LIM homeobox 2) gene, encoding a transcriptional regulator.

Damiani et al. showed that the specific removal of the *DICER1* gene from the mouse retina resulted in an inability of photoreceptors to respond to light; these structures also underwent morphological changes in the form of photoreceptor rosettes progressing to degeneration of the aging retina [58]. These effects were associated with downregulation of most miRNAs in the retina. Lumayag et al. reported that an inactivation of the miR-183/96/182 cluster, expressed at high levels in the retina, was associated with defects in photoreceptors resulting in progressive retinal degeneration [52]. Furthermore, silencing the cluster resulted in changes in retinal gene expression, including genes essential for photoreceptor morphogenesis and phototransduction.

## Other aspects of DICER1 deficiency/*Alu* RNA accumulation in the retina

Translocation of *Alu* transposons can affect genes with different consequences - from a complete inactivation to an increase in their expression. It was reported that homozygous *Alu* insertional variants in the angiotensin-converting enzyme (ACE) gene occurred almost five times more frequently in the general population than in a population of dry AMD patients [59]. The *ACE* gene is expressed both in RPE cells and the neural retina. Therefore, *ACE* insertion of *Alu* elements can exert protective effects against AMD. This insertion, although in an intronic region of the *ACE* gene, results in a lower amount of its product, suggesting the silencing function of the *Alu* element. Interestingly, no association between the *Alu* polymorphism in the *ACE* gene and wet AMD was observed in that study. This is an apparent discrepancy with results associating the homozygous *Alu* genotype with proliferative diabetic retinopathy, but the sources of new vessel in AMD and diabetic retinopathy are different [60].

Lutein is known as an AMD preventive compound and it was shown to improve the viability of ARPE-19 cells and reduce the amount of *Alu* RNA in cells challenged with hydrogen peroxide [61]. However, lutein had no impact on DICER1 level as evaluated by immunoblotting, but this does not exclude DICER1 involvement in the observed effects, as its activity was not measured in that experiment. Lutein is known to exert the protective action in the eye mainly by absorption of UV light and quenching free radicals [62]. Therefore, lutein might ameliorate oxidative stress in the examined cells preventing downregulation of the DICER1 gene that is in line with the suggestion of Kaneko et al. that oxidative stress is an initial drive to trigger a cascade of events leading to DICER1 inhibition/*Alu* accumulation.

Yan et al. observed a diurnal variation of DICER expression in various tissues, including the mouse retina, in which the peak expression was observed at Zeitgeber Time 21 [63]. Next, these authors evaluated whether the diurnal pattern of DICER expression was changed with age, comparing it in mice at 2 and 24 months. The aged mice showed a reduced DICER1 expression. In addition, changes in the diurnal pattern of expression of miR-146, involved in the pro-inflammatory reaction and miR-125a-5p, involved in apoptosis, were observed in bone marrow nucleated cells in aged mice. Transfection of human retinal endothelial cells (HRECs) with *Alu*-expressing plasmid resulted in an increased activity of caspase-3. Therefore, the age-related loss of DICER1 can contribute to retinal disorders by affecting not only RPE cells, but also HRECs.

Liu et al. observed the expression profile of miRNAs in a mouse model of oxygen-induced retinopathy (OIR) and noted that 23 of them were either down- or up-regulated in OIR retinas [56]. The observed decrease in miRNA expression was in line with the decreased expression DICER1 and the corresponding increase in that of *Alu* RNA, supporting the general conclusion for the involvement of *Alu* RNA excess in retinal disorders induced by DICER1 impairment.

## Conclusion and perspectives

The results and their interpretation presented in this review show a possible pathway of the involvement of DICER1 deficiency and *Alu* accumulation in AMD pathogenesis. Oxidative stress, a major AMD risk factor, downregulates the DICER1 gene in the retina, resulting in decreased amount and activity of its product. This leads to *Alu* accumulation that disrupts mitochondrial homeostasis, releasing mtDNA into cytosol and NLRP3 activation, important in AMD pathogenesis. It is not clear how *Alu* RNA accumulation affects mitochondrial integrity and this problem should be addressed in perspective mechanistic research.

Caspase-1 may be critical element in DICER1 deficiency-related AMD pathogenesis as it was reported that an *Alu* excess did not induce RPE degeneration in mice deficient in its activity. The release of oxidized mtDNA, a consequence of programmed cell death, can also induce the NLRP3 inflammasome. However, it was revealed that mitochondrial apoptosis is not needed for NLRP3 activation and that caspase-8 might play an important, apoptosis-independent regulatory role in this process [64]. Caspase-1 can induce mitochondrial disintegration associated with ROS overproduction [64]. Moreover, caspase-1 has been reported to cleave Parkin, a crucial protein of mitophagy, which is an essential element of mitochondrial quality control [65]. Therefore, there is a coupling between inflammasome activation and mitochondrial damage - the damage can activate the inflammasome and mature pro-caspase-1 inducing pathways leading to mitochondria breakdown. We and others have demonstrated that the cellular reaction to DNA damage, including damage to mtDNA, is impaired in AMD patients [4, 66, 67].

Is DICER1 deficiency-induced *Alu* RNA accumulation in the retina of clinical significance? First of all a systemic analysis of this effect or its consequences could be performed as its proposed mechanism underlying its involvement in AMD pathogenesis does not seem to be limited to the retina and could be reflected in other mitochondria-rich and oxidative stress-exposed organs. Possible systemic markers associated with this effect could be tested to assess their suitability in predicting and evaluation of AMD progress, treatment and follow-up as well as their personalization. Although there is not possible to evaluate these possible markers in the retina in clinical practice, DICER1 deficiency/*Alu* RNA accumulation could be considered to be evaluated in aqueous humor and vitreous body. This issue should be addressed in further research to answer the general question whether the discovery of the effect of DICER1 deficiency/*Alu* accumulation in AMD retinas bring us close to developing new therapeutic strategies against AMD.

**Figure 4.**
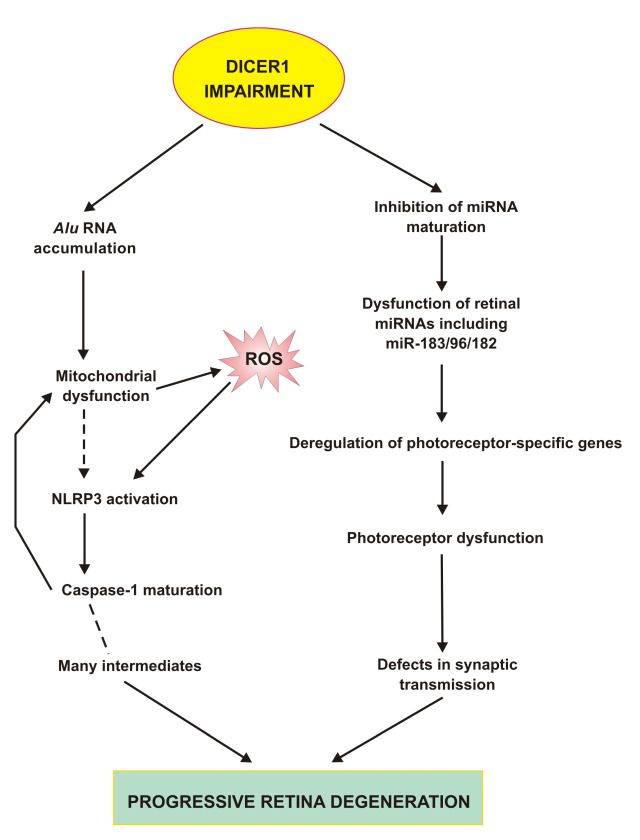
**Impairment in DICER1 in the retina may result in the accumulation of *Alu* transcripts and disturbances in miRNA biogenesis**. *Alu* RNAs can induce mitochondrial dysfunction in retinal pigment epithelium (RPE) leading to excessive ROS production resulting in NLRP3 inflammasome activation and caspase-1 maturation, which can increase mitochondrial damage. NLRP3 activation is associated with the production of many intermediates and eventually leads to RPE cells degradation and death. Disturbed miRNA biogenesis can result in the deregulation of expression of many genes involved in retinal homeostasis in both RPE and neural retina and the miR-183/96/182 cluster belongs to the most important elements of that regulation, but as far as we are aware, all aspects of miRNA regulation in the retina are still far from clear. Only some aspects of dysregulated miRNAs in the neural retina are presented.

DICER1 deficiency could inhibit the maturation of miRNA and therefore this could change the expression of many genes important for RPE cells and the retina in general [68]. The best known is the function of the miR-183/96/182 cluster, which regulates photoreceptors and their synaptic transmission. The inactivation of the miR-183/96/182 gene leads to a progressive retinal dysfunction and degeneration (Fig. 4) [52]. Again, clinical significance of DICER1 deficiency-related changes in miRNA depends on the systemic consequences of this effect occurring in the retina. miRNA may be quantified in the aqueous humor [69]. One of the most intriguing aspects of the involvement of DICER1 deficiency in AMD pathogenesis is the clear separation of consequences of the secondary effects of this deficiency - *Alu* RNA accumulation and miRNA dysregulation into GA and wet AMD, respectively. This issue might be addressed in a single comparative analysis.

Another aspect of the role of DICER1 in AMD pathogenesis is its circadian regulation in the retina. It has been observed that aging resulted in either the loss of circadian rhythm of DICER1 or its decreased expression and changes in diurnal pattern of DICER1 were displayed in both types of RNAs, whose biogenesis it regulates: miRNAs and *Alu* RNAs [63]. However, the role of miRNA could be considered in a broader context of the role of epigenetic regulation in AMD pathogenesis, specifically miRNA-lncRNA (long non-coding RNA) interaction.

In summary, DICER1 deficiency in AMD may play an important role in the pathogenesis of both main forms of the disease: dry and wet AMD. *Alu* RNA accumulation induced by DICER1 impairment can induce a significant pathway of GA pathogenesis, whose clinical significance should be further explored.
